# Effectiveness of shading and linear perspective cues in eliciting three-dimensional perception of bidimensional images in dogs

**DOI:** 10.1007/s10071-025-02026-0

**Published:** 2025-12-29

**Authors:** Anna Broseghini, Valeria Bevilacqua, Cécile Guérineau, Paolo Mongillo, Lieta Marinelli

**Affiliations:** Department of Comparative Biomedicine and Food Science, Università degli Studi di Padova, Viale dell’Università 16, Legnaro, 35020 PD Italy

**Keywords:** Vision, Depth perception, Three-dimensionality, Pictorial cues, Linear perspective, Shading, Dogs

## Abstract

Three-dimensionality perception relies on the integration of various depth cues, with monocular pictorial cues playing a key role in two-dimensional representations. Previous research indicates that dogs are sensitive to a combination of shading and linear perspective when perceiving three-dimensionality, yet the individual contributions of these cues remain unclear. The aim of the present study was to disentangle the specific contribution of shading and linear perspective in promoting three-dimensional perception in dogs. In a series of four experiments involving 120 dogs, subjects were presented with a ball rolling on an apparatus and either falling inside a real hole (control condition) or keeping rolling over a depicted hole (test condition). Experiments 1 and 2 assessed the individual contribution of linear perspective and shading in dogs’ perception of three-dimensionality; Experiment 3, as a replica of previous findings, investigated the combination of both cues; Experiment 4 explored the role of more intense shading. In a violation of expectation paradigm, dogs showed no sensitivity to the pictorial cue of linear perspective or low shading level alone. Conversely, the combination of linear perspective and shading, as well as intense shading alone, successfully elicited three-dimensional perception. These results confirm that dogs can successfully integrate multiple pictorial cues to perceive three-dimensionality from two-dimensional stimuli. The perception of three-dimensionality can also occur through single cues, but only if the cue is sufficiently intense, at least in the case of shading.

## Introduction

Perception of depth refers to the extraction of distance cues from a visual scene, computing depth order, understanding which surfaces lie in front of others, and depth intervals, using relative disparities to judge how far apart objects are (Anzai and DeAngelis 2010). Three-dimensional perception requires the integration of different depth cues into a full representation of the three-dimensional structure of visual scenes (Dövencioğlu et al. 2013). The visual system obtains depth information through two types of cues: binocular cues, which rely on input from both eyes, and monocular cues which provide three-dimensionality and distance information even if the scene is viewed with only one eye (Teittinen 1993). Among monocular cues there are the pictorial cues of linear perspective and shading, which are the focus of the present study. Linear perspective refers to the apparent convergence of parallel lines on a single point as they recede away from the viewer and towards the horizon. Shading provides depth cues through changes in light and shadow, reflecting the orientation of the surface relative to the light source (Palmer 1999). Along with others, these cues help make sense of the three-dimensional structure of visual scenes and allow the brain to construct a three-dimensional interpretation even from flat, two-dimensional representations (Cook et al. 2008).

The animal’s use of pictorial cues has great ecological relevance. For instance, manipulation and perception of pictorial cues were shown to play a role in sexual selection (Kelley and Endler 2012) and anti-predator strategies (Rowland 2008). Understanding how animals use pictorial cues is also important in experimental contexts, in the field of cognitive and behavioural sciences, where animals are often presented with 2D images that simulate three-dimensional environments ( e.g., Cabe 1976; Dérian et al. 2012; Luden 1977; Trillmich 1976). A clear understanding of the role of pictorial cues in the perception of two-dimensional images would be fundamental for the accurate interpretation of results. Knowledge regarding the animal’s reliance on different types of pictorial cues is uneven. Many studies explored the ability of animals to extrapolate three-dimensional information from shading. It was shown, for instance, that shading allows pigeons and chicks to differentiate between concave and convex surfaces (Cook et al. 2012; Hershberger 1970). Young chimpanzees can transfer reaching responses from three-dimensional convex objects to two-dimensional convex models, using pictorial depth defined by shading information (Imura and Tomonaga 2003). The use of shading also extends to more distant taxa. For instance, cuttlefish adjust the shading of a specific body pattern when placed on backgrounds containing shaded circles or 3D hemispheres, suggesting they can interpret shading as an indicator of three-dimensional shapes (Zylinski et al. 2016). In summary, the use of shading to infer three-dimensionality is well supported and widespread in the animal kingdom.

Conversely, the effect elicited by linear perspective was relatively less studied in animals, and mainly indirectly, by assessing susceptibility to the Ponzo or the Corridor illusion, where the same lines or objects seem of different size when placed within converging lines or a corridor-like background (horses: Timney and Keil 1996; monkeys: Bayne and Davis 1983; pigeons: Fujita et al. 1991, dogs: Byosiere et al. 2017, 2018; baboons: Barbet and Fagot 2002). However, these illusions require more than mere ability to infer three-dimensions from perspective, for example size estimation skills, hence results of these studies cannot be taken as evidence in support of or against animal’s ability to use perspective towards the perception of depth.

The study of dogs’ visual perception is of great interest, both because visual tasks are the most common method for assessing canine cognition (Byosiere et al. 2018) and because dogs have proven to be an ideal model for comparative studies, given their similarities to humans in visual perception (Looke et al. 2024). Yet, the understanding of the ability of dogs to perceive depth and three-dimensionality is still scant. Dogs have a binocular visual field with an estimated overlap between 30° and 60°, considerably smaller than the 140° overlap of humans (Miller and Murphy 1995). Studies on retinal ganglion cells suggest dogs’ depth perception may be limited, as the area optimized for high-quality depth perception seems smaller than what expected on the basis of the visual field overlap alone (Miller and Murphy 1995; Peichl 1992). One study using fMRI has shown that dogs do not automatically generalize between two- and three-dimensional stimuli, and that reward-processing areas and parietal regions of the cortex responded selectively to the stimulus type (2D or 3D) on which the dog has been trained (Prichard et al. 2021). Due to dogs’ cephalic morphological variation and associated degree of lateralization of the eyes, reliance on binocular disparity might largely differ between breeds (Barber et al. 2020). On the other hand, the use of monocular cues is unaffected by head morphology, making the exploration of these mechanisms more suitable for comparative studies of visual perception. Despite their suitability, the role of monocular cues has rarely been studied.

One study demonstrated that dogs could recognize 3D stimuli and their rotated versions when displayed digitally, supporting their ability to process three-dimensional information even when presented in 2D representations (Siniscalchi et al. 2023). The study however does not inform on what specific cues were used by dogs to infer three-dimensionality. Byosiere and colleagues found no susceptibility of dogs to the Ponzo illusion (Byosiere et al. 2018), which requires the ability to perceive the pictorial cues of linear perspective. However, as stated above, the task is not only a simple visual task but requires the estimation of item size based on linear perspective rules, preventing us from attributing the lack of susceptibility to the illusion to an inability to use linear perspective as a depth cue. A recent study found that the combined presence of linear perspective and shading was able to elicit the perception of three-dimensionality in bidimensional images in dogs (Broseghini et al. 2024). Presented with either a ball rolling towards and falling into a real hole (control) or a ball rolling over a hole depicted in a bidimensional image (test), dogs showed a surprised reaction at the latter, unexpected event, suggesting that the pictorial cues of shading and linear perspective were effective in eliciting the perception of three-dimensionality. However, since both pictorial cues were represented in the two-dimensional image, this study does not allow to disentangle the individual contribution of linear perspective and shading. For this reason, the aim of the present study was first to investigate independently the role of linear perspective (Experiment 1) and shading (Experiment 2). Adopting the same methodology used by Broseghini and colleagues, we isolated each pictorial cue to determine its specific contribution in eliciting depth and three-dimensionality perception. Considering the negative results, two further experiments were performed. In Experiment 3 we aimed to replicate the results of Broseghini and colleagues, assessing the robustness of the previous findings, while Experiment 4 explored the effect of increasing the level of the shading cue, addressing the possibility that a potentially low-level shading may have led to absence of responses when presented alone.

## Materials and methods

### Subjects

For this study, 125 companion dogs were initially involved. The dog owners were recruited on a voluntary basis and were either students at the University of Padua or recruited *via* the Laboratory of Applied Ethology database of volunteers. The criteria for dog selection included being adult and in good health, without any known vision impairments, showing ease of adaptation and interaction in new environments, and having an eye-level height between 35 and 100 cm when in a sitting position. The final sample consisted of 120 dogs (69 females, 51 males; average age ± SD = 4.4 ± 3.2 years; 51 Mixed-breed, 6 Border Collies, 5 Australian Shepherds, 5 German Shepherds, 5 Labrador Retrievers, 5 Lessinia and Lagorai Shepherds, 4 Jack Russels, 3 Cocker Spaniels, 3 French Bulldogs, 3 Lagotto Romagnolo, 2 Australian Kelpies, 2 Maltese, 2 Poodles, 2 Czechoslovakian Wolfdogs, 2 Shiba Inu, 1 Beagle, 1 Bolognese, 1 Chihuahua, 1 Dachshund, 1 English Bulldog, 1 English Setter, 1 English Springer Spaniel, 1 German Pointer, 1 Golden Retriever, 1 Irish Setter, 1 Italian Bloodhound, 1 Pomeranian, 1 Portuguese Water Dog, 1 Flat-Coated Retriever, 1 Spanish Greyhound, 1 Pitbull, 1 Rottweiler, 1 Shih Tzu, 1 Vizsla, 1 Weimaraner). The remaining five dogs were excluded due to technical problems or their lack of attention to the ball at the start of the trial. As the experimental design relied on obtaining valid data from the two conditions for any given subject, if only one of the two trials was compromised, both trials were excluded from analysis for that dog. The 120 dogs were divided into four sub-samples, in which 30 dogs were allocated to one of the four experiments.

### Experimental setting

**Fig. 1:**
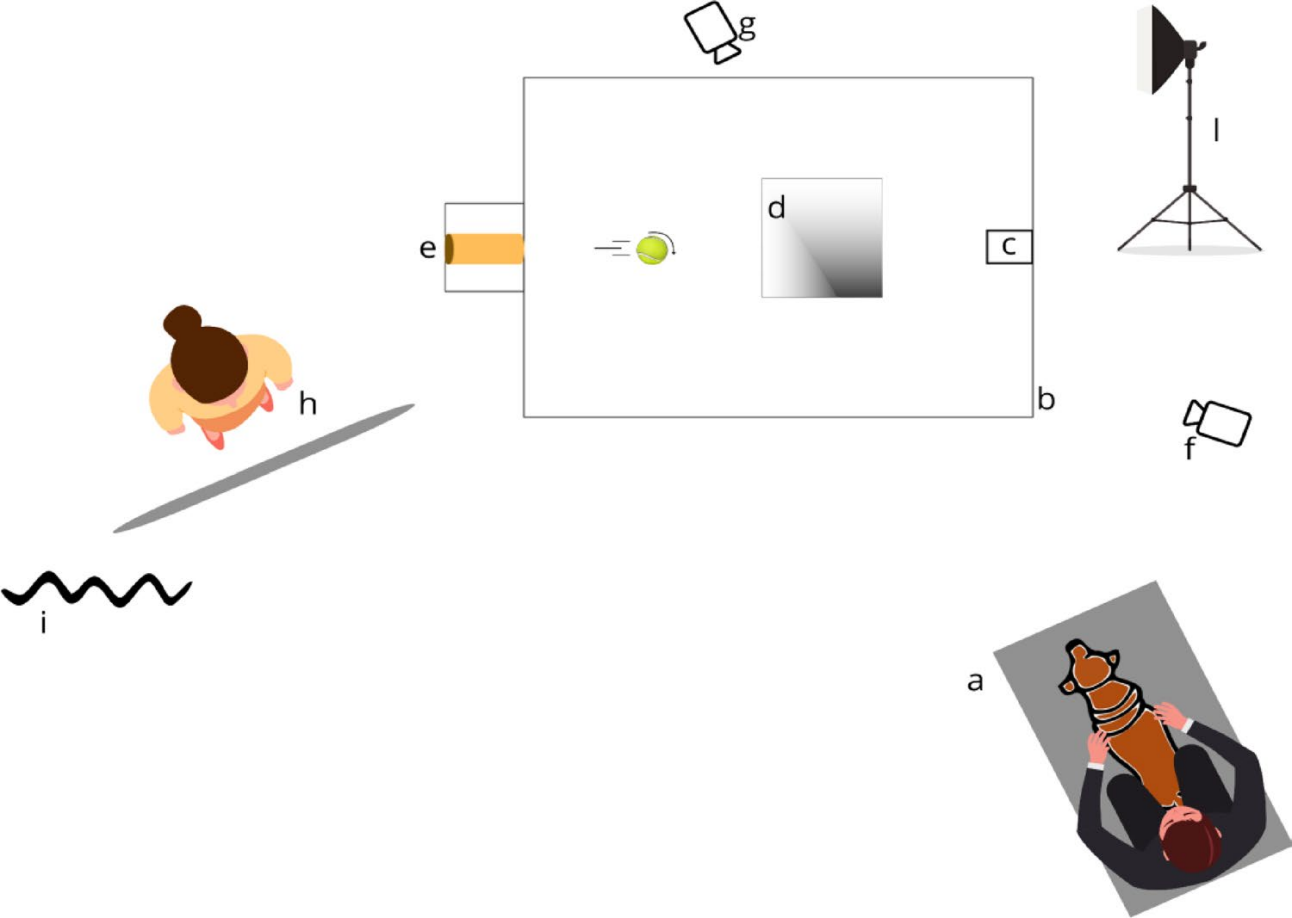
Depiction of the experimental setting: the dog held by the owner positioned on the platform (**a**), the experimental apparatus of Experiment 4 with the ball rolling on it (**b**), the small box at the end to collect the ball when the hole was closed (**c**), the illusory/real hole (**d**), the paper tube through which the ball is dropped (**e**), the camera directed at the apparatus (**f**), the camera recording the dog’s eyes (**g**), the experimenter placing the ball into the tube, concealed behind the opaque panel (**h**), the curtain used to hide the apparatus (**i**), the spotlight to illuminate the apparatus (**l**). The second experimenter giving instructions to the caregiver, and the ceiling camera recording the dog’s head are not shown. The elements in the figure are not drawn to scale.

The test was carried out in an experimental room (6.0 × 3.6 m). The experimental setup was identical to the one presented in the previous study (Broseghini et al. 2024) and is visible in Fig. 1.

A blanket-covered platform was provided as a seating area for the dog and the caregiver, and its axis was aligned with the direction of the depicted hole image. The platform was made of adjustable polyurethane foam elements to raise the dog’s eye level to approximately 100 cm when sitting, and it was positioned 240 cm away from the apparatus. The apparatus consisted of a plastic board (152 × 102 × 1 cm) elevated 25 cm above the floor. A hole (30 × 30 cm) was cut into the board, with the option to either leave it open (“Real hole” condition) or cover it with a panel featuring a photograph of the hole, creating the “Depicted hole” condition. The experimental setup included a 30° inclined paper tube positioned to allow a ball to roll across the board, with transparent fishing lines guiding its movement to maintain a straight path. An opaque panel concealed the experimenter, whose role was to place the ball in the tube. A white curtain covered the apparatus prior to the start of the test. Three video cameras were used to record the experiment (two AVer TR311HWV2, Taipei, Taiwan, and one Canon XA20, Tokyo, Japan): one overhead to monitor the dog’s head orientation, one directed at the apparatus, and one focused on the dog’s eyes (with the possibility to turn the infrared mode on in case of Exp. 4) to confirm alignment with head movements.

**Fig. 2:**
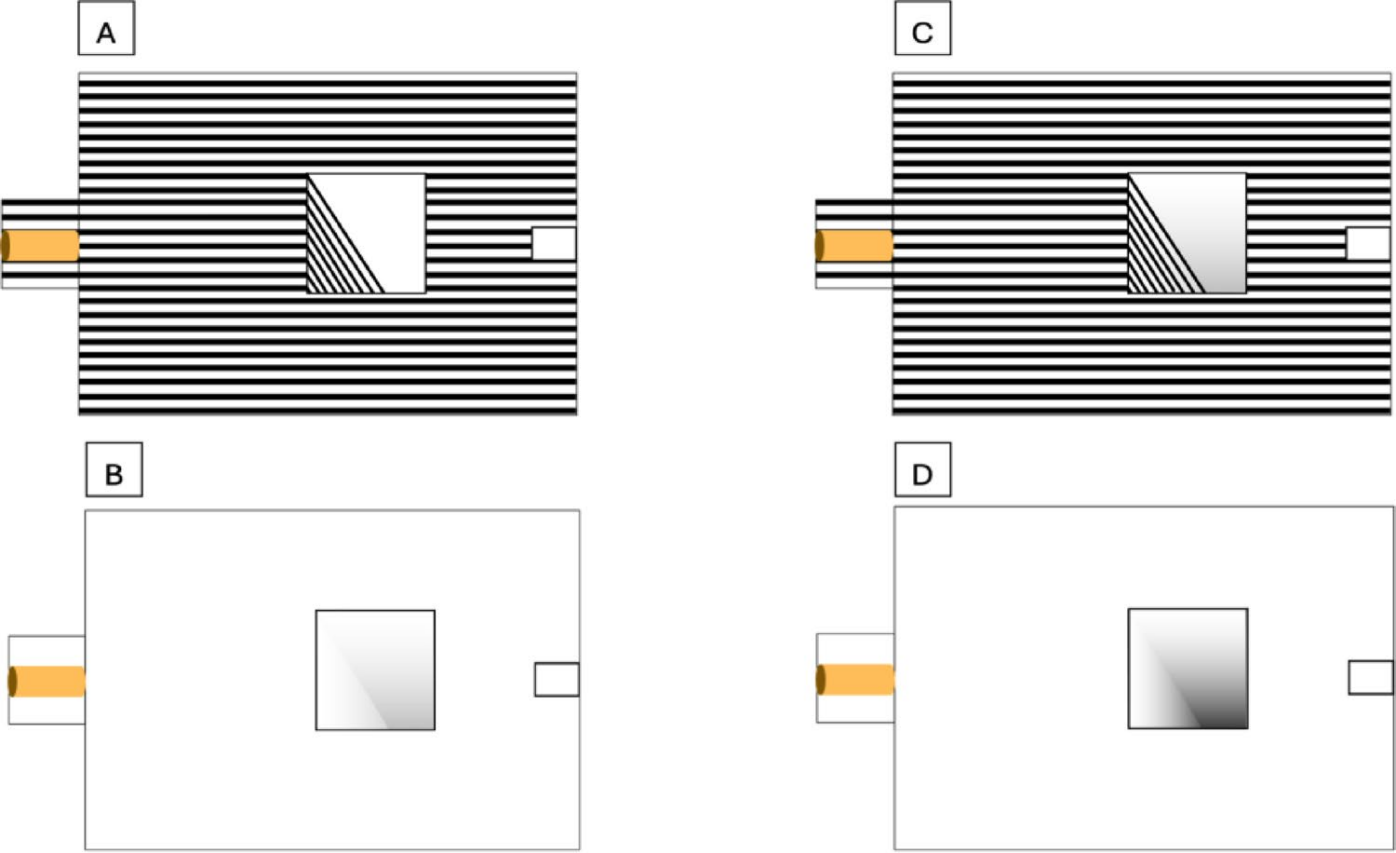
Representation of the experimental apparatus used in each of the four experiments, i.e., with only linear perspective (**A**; Experiment 1), only shading (**B**; Experiment 2), with both pictorial cues (**C**; Experiment 3) and only a higher level of shading (**D**; Experiment 4)

Initially, two experiments were conducted, each isolating one of the two pictorial cues which were used in combination by Broseghini and colleagues (2024); thus, Experiment 1 featured only linear perspective (Fig. 2A), while Experiment 2 included only low contrast shading (Fig. 2B). Two additional experiments were implemented: the exact replica of the stimulus used by Broseghini and colleagues (2024) with both pictorial cues (Fig. 2C, Experiment 3) and one experiment where only a high level of shading was presented (Fig. 2D, Experiment 4). In the latter, not only shadows of the image covering the hole were made more intense/contrasted, but the apparatus was intentionally lit by a photography lighting kit (ESDDI Softbox 900 W, Shenzhen ESDDI Technology Co., Ltd, Shenzhen, China), while the main lights of the lab were turned off, to increase the luminance contrast between the apparatus and the hole. Apart from lighting, the experimental setting for each experiment remained identical.

### Procedure

The procedure followed for the four experiments in this study was identical to that described in the previous work by Broseghini and colleagues (2024). Briefly, before the experiment started, the height of the sitting platform was adjusted according to the dog’s height. Owners were instructed to sit on the platform with the dog between their legs, gently holding the dog still throughout the test without interacting or influencing its behaviour, and to look down and not at the ongoing test. An experimenter helped the dog and owner to settle, and then opened the curtain to reveal the apparatus; then another experimenter, hidden behind the panel on the left of the apparatus, attracted the dogs’ attention to a ball, using movements and verbal cues. If the dog did not focus on the ball within 10 s, it was excluded from the test. Dogs’ attention was monitored by the experimenter through a small slit in the panel, covered with a one-way curtain that allowed her to see the dog without being seen. Once the dog’s attention was secured, the ball was released into the tube and either disappeared by landing in the hole (‘Real hole’ condition) or reached the box at the end of the apparatus (‘Depicted hole’ condition). Nothing else happened for the next 30 s, after which, the experimenter escorted the owner and the dog out of the testing room. After a fifteen-minute break, the same procedure was repeated, presenting the dog with the second condition, being either the control or test. The order of presentation of test and control conditions was counterbalanced among dogs within each experiment.

### Data collection and analysis

Data about the dog’s orientation was collected with Observer XT (version 12.5, Noldus, Wageningen, The Netherlands), with a continuous sampling technique from the moment the ball was released into the tube until 30 s after the ball disappeared. Based on the tape lines on the floor, the orientation of the dog’s head was coded as toward the ‘beginning of the apparatus’ (from the panel hiding the experimenter to the beginning of the hole), the ‘end of the apparatus’ (from the beginning of the hole to the end of the apparatus). The camera focusing on the dog’s eyes was used to confirm the alignment with head movements and the orientation of the dog’s look. The coders only had access to the videos that pointed towards the dog's head and eyes, so they were blind to the experimental condition. To assess interobserver reliability, 30% of the videos of each experiment were coded by a second person. Interobserver reliability was very good for all data collected, ranging from 0.78 to 0.97. To determine whether the dog’s attention was equally attracted by the rolling ball both in the test and control conditions of each Experiment, paired t-tests were conducted to compare the dog’s looking time at the beginning of the apparatus from the moment the ball left the tube until it reached the margin of the hole.

The percentage of time the dogs spent looking at the end of the apparatus while the ball was travelling through that area in the test condition was also calculated. According to the violation of expectation paradigm, if dogs are sensitive to the pictorial representation of the hole, they should be surprised when the ball keeps rolling without falling into it. Surprise is indicated by increased attention toward the area where the unexpected event occurs, compared to a control condition (Winters et al. 2015). In our procedure, the potential expectation violation took place in the ‘end of the apparatus’ area. Therefore, we used a Generalized Estimating Equation (GEE) model to analyse the total duration of the dog’s orientation towards the area ‘end of the apparatus’ during the 30 s after the ball fully disappeared, between the control and test conditions within each experiment. The looking time at the end of the apparatus was included as a dependent variable in the model. The experimental condition, trial order and interaction between these two factors were included as independent variables. The identity of the dog was included as a random effect to account for repeated measurements in the same subject. The same model was also used to analyse the duration of the dog’s orientation towards the other area, including looking at ‘beginning of apparatus’ as dependent variable. As the majority of the data were not normally distributed, we employed a Tweedie distribution with a log link function in the GEE model to assess the dogs’ looking time at the different parts of the setting in the 30 s after the disappearance of the ball.

All statistical analyses were performed with SPSS (v. 28, IBM, Armonk, NY). The level of statistical significance was set at 0.05. All looking data are presented as mean values ± SD, unless otherwise stated.

## Results and discussion

### Experiment 1 - Linear perspective

#### Results

The mean total duration of “Depicted hole” trials was 32.0 ± 0.3 s and for the “Real hole” trials it was 31.0 ± 0.1 s. Dogs were oriented towards the ball travelling in the area “beginning of apparatus” for 75.7 ± 7.3% of the 1.0 s time. The t-test confirmed no difference in looking time towards the beginning of apparatus for the two experimental conditions (Depicted hole condition = 0.8 ± 0.1 s; Real hole condition = 0.8 ± 0.1 s; t = 0.661, df = 29, *p* = 0.514, Cohen’s d = − 0.121). In the Depicted hole condition, dogs were oriented towards the “end of apparatus” when the ball travelled from the hole to the box for 93.7 ± 24.1% of the time.

**Fig. 3:**
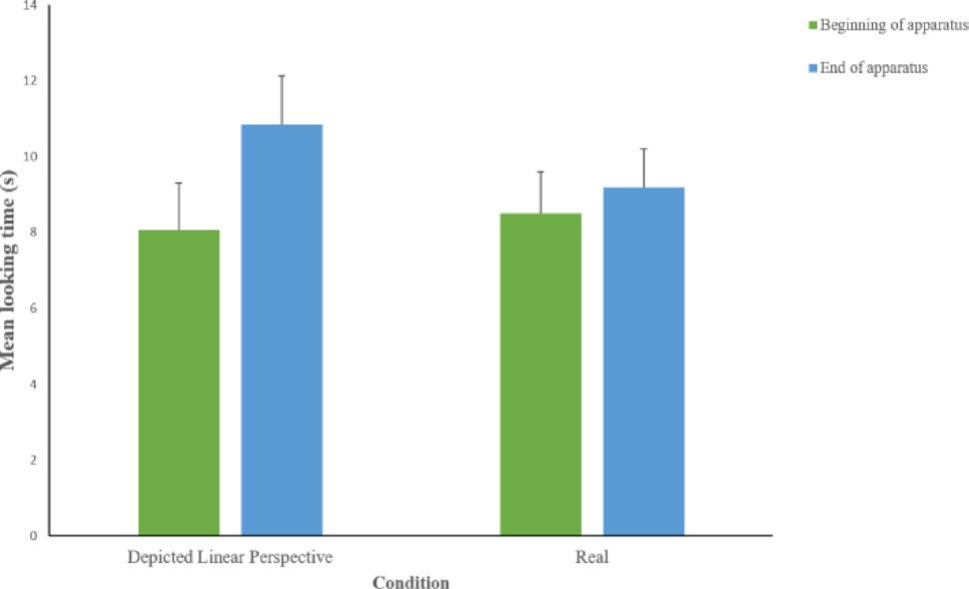
Mean looking time (± SE) paid by dogs to different parts of the experimental setting and elsewhere, in the Depicted hole with Linear Perspective and Real hole conditions, during the 30 s after the ball has disappeared

Data on the looking times at the different areas (“beginning of apparatus,” “end of apparatus”) in the 30 s after the disappearance of the ball, for both experimental conditions, are shown in Fig. 3.

Dogs’ looking time to the “end of the apparatus” was not affected by condition (Wald Chi-square = 2.079, *p* = 0.149), order of presentation (Wald Chi-square = 3.729, *p* = 0.053), or their interaction (Wald Chi-square = 0.063, *p* = 0.802). The same non-significant results were found for the looking time at the “beginning of apparatus” for the condition (Wald Chi-square = 0.129, *p* = 0.719), order of presentation (Wald Chi-square = 1.046, *p* = 0.306), and interaction between the two (Wald Chi-square = 0.019, *p* = 0.892).

#### Discussion

The first experiment investigated the role of linear perspective towards the perception of three-dimensionality. The lack of a surprise reaction to the unexpected event suggests that dogs are unable to perceive the three-dimensionality of a two-dimensional image using linear perspective alone. Linear perspective was represented by alternating black and white lines, which formed a visually distinct, high-contrast pattern. Alternating black and white visual patterns have been effectively used to promote depth perception, for instance in the famous checkerboard patterns of the “Visual cliff” (Gibson and Walk 1960). Also, even after the ball was no longer visible, dogs kept a high level of attention to the experimental apparatus, comparable to that observed by Broseghini and collaborators (2024). Therefore, neither a lack of attention nor a low salience of the stimulus is likely to justify the negative results and suggests that dogs might not be susceptible to perspective, at least in the form presented in the current experiment. This result seems to be in line with the lack of susceptibility to the Ponzo illusion in dogs observed by Byosiere and colleagues (Byosiere et al. 2018), although, as mentioned above, this task involves not only perception but also size estimation, making the comparison with the Ponzo illusion somewhat limited.

### Experiment 2 – Shading

#### Results

The mean total duration of “Depicted hole” trials was 31.7 ± 0.3 s, and for the “Real hole” trials was 31.0 ± 0.1 s. Dogs were oriented towards the area “beginning of apparatus” for 82.3 ± 16.0% of the 1.0 s time, when the ball was travelling in this area. There was no difference in looking time towards the “beginning of apparatus” area for the two experimental conditions (Depicted hole condition = 0.8 ± 0.2 s; Real hole condition = 0.8 ± 0.2 s; t = − 0.029, df = 29, *p* = 0.977, Cohen’s d = −0.005). In the Depicted hole condition, dogs were oriented towards the final part of the apparatus when the ball travelled in this area for 90.6 ± 20.0% of the time.

**Fig. 4:**
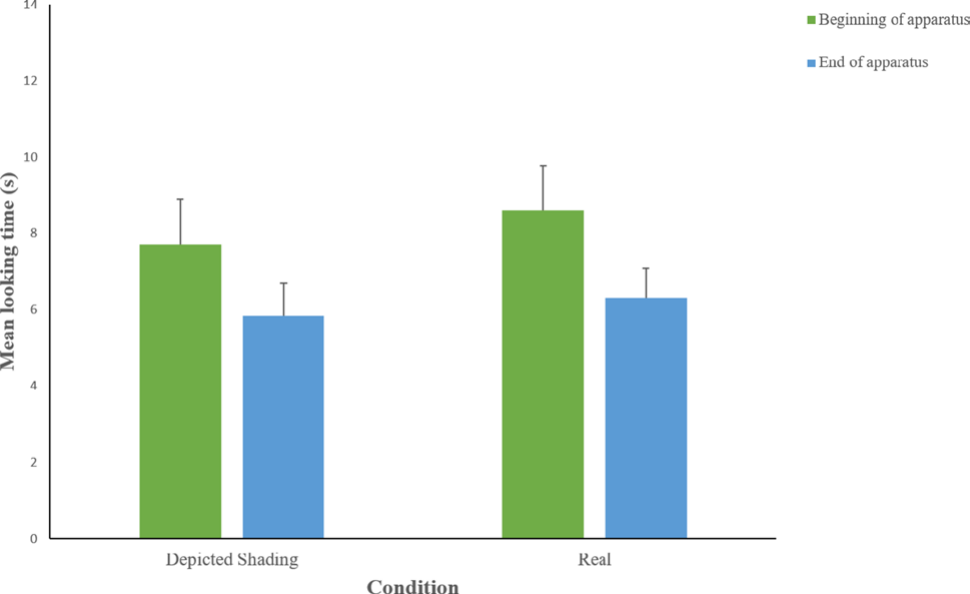
Mean looking time (± SE) paid by dogs to different parts of the experimental setting and elsewhere, in the Depicted hole with shading and Real hole conditions, during the 30 s after the ball disappeared

Data on the looking times at the different areas (“beginning of apparatus,” “end of apparatus”) in the 30 s after the disappearance of the ball, for both experimental conditions, are shown in Fig. 4.

Dogs’ looking time at the “end of the apparatus” in the 30 s after the disappearance of the ball was not affected by condition (Wald Chi-square = 0.957, *p* = 0.328). The order of presentation had an effect on the looking time at the “end of the apparatus” (Wald Chi-square = 4.968, *p* = 0.026, Cramer’s V = 0.957), being higher in the first trial (estimated mean ± std. error = 6.8 ± 0.7 s) than in the second trial (estimated mean ± std. error 5.1 ± 0.8 s). The interaction between these variables did not affect the looking time at the “end of apparatus” (Wald Chi-square = 2.408, *p* = 0.121). Looking at the “beginning of apparatus” was not affected by condition (Wald Chi-square = 0.640, *p* = 0.424), order of presentation (Wald Chi-square = 0.000, *p* = 0.989), and interaction between the two (Wald Chi-square = 0.004, *p* = 0.952).

#### Discussion

In the second experiment, when only shading was presented, the experimental condition did not affect the looking time towards the area where the unexpected event occurred, suggesting a failure of shading to individually promote the perception of three-dimensionality in dogs. The absence of susceptibility to the shading cue alone found in the present experiment, together with the absence of susceptibility to linear perspective found in Experiment 1, combined with the effect previously revealed by the integration of both cues (Broseghini et al. 2024), suggests that linear perspective and shading together are necessary to elicit the perception of three-dimensionality of 2D images. In order to confirm this hypothesis and to strengthen the previous results, we decided to repeat the experiment involving the presentation of both cues (see Experiment 3).

However, the result of the present experiment was unexpected, as shading is widely recognized as a key cue for depth perception in many species, playing a critical role in the interpretation of three-dimensionality in non-human animals (Cook et al. 2012; Hershberger 1970; Imura and Tomonaga 2003; Zylinski et al. 2016). One possible explanation for this ineffectiveness is that the intensity of the cue was too low. In fact, to provide continuity with the experiment by Broseghini and colleagues (2024) we kept the identical shading characteristics of the stimulus used in such experiment, which may have compromised its effectiveness as a single depth cue in this context. To test this hypothesis, we implemented a fourth experiment, increasing the intensity of shading (see Experiment 4).

In the present experiment a trial order effect emerged, regardless of condition. It is not unusual in dogs’ visual attention tasks to find an effect of the order of presentation (Lõoke et al. 2023). This is likely due to an increase of familiarization with the task and a decreased interest of subjects towards stimuli that are of little salience or relevance, as also suggested by the relatively low attention paid by subjects to the apparatus overall. The small sample size might also have contributed to the emergence of order effects, in the absence of stronger factors affecting attention (e.g. surprise).

### Experiment 3 - linear perspective and shading

#### Results

The mean total duration of “Depicted hole” trials was 31.6 ± 0.3 s, and for the “Real hole” trials was 31.0 ± 0.1 s.

Dogs’ orientation toward the ball travelling in the area “beginning of apparatus” was 97.0 ± 6.4% of the 1.0 s in Experiment 3. The looking time towards the “beginning of apparatus” area for the two experimental conditions showed no difference in a t-test (Depicted hole condition = 1.0 ± 0.1 s; Real hole condition = 1.0 ± 0.1 s; t = − 0.880, df = 29, *p* = 0.386, Cohen’s d = −0.161). Dogs were oriented toward the final part of the apparatus when the ball travelled from the hole to the box for 76.3 ± 21.5% of the time, in the Depicted hole condition.

**Fig. 5:**
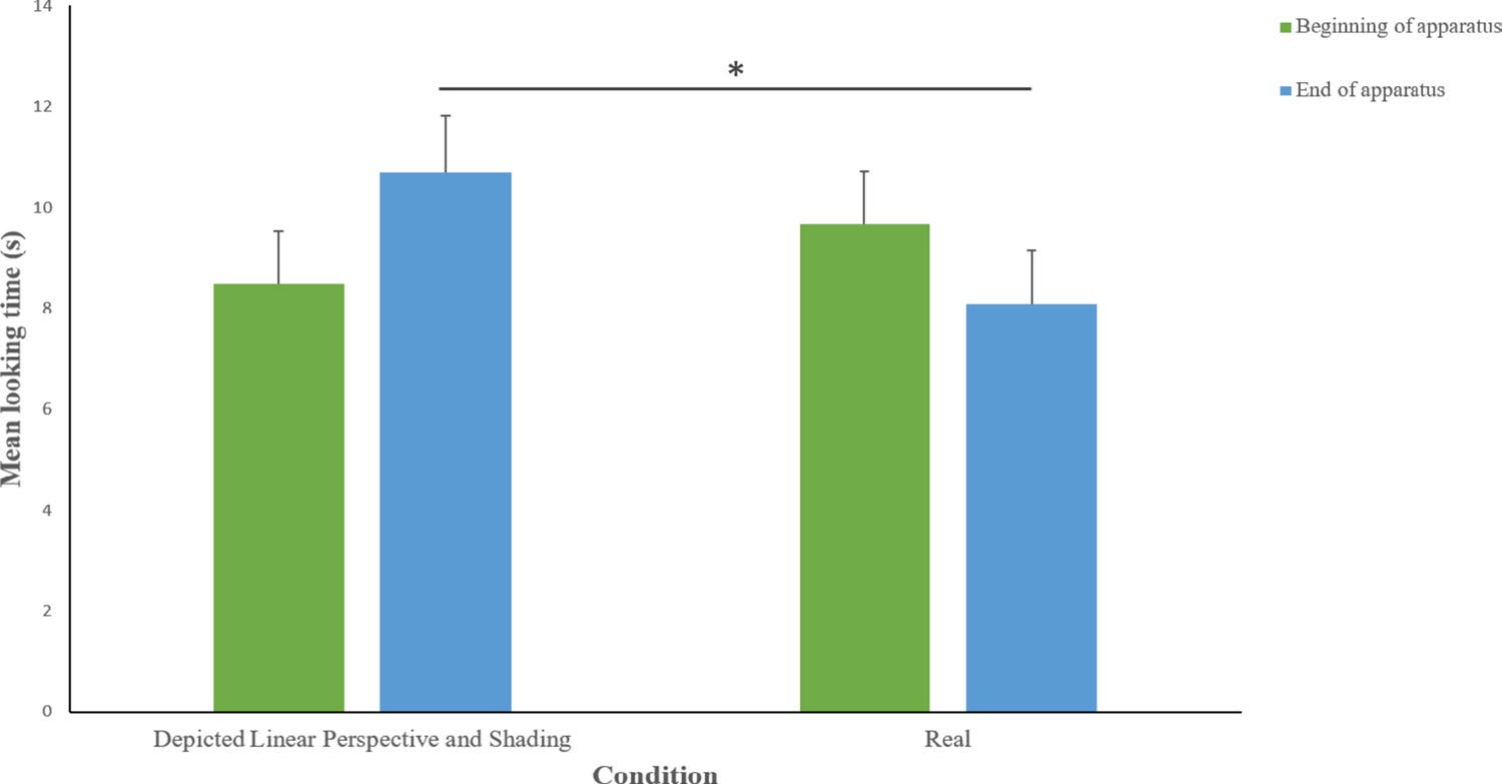
Mean looking time (± SE) paid by dogs to different parts of the experimental setting and elsewhere, in the Depicted hole with linear perspective and shading cues, and Real hole conditions, during the 30 s after the ball disappeared. Line with asterisks indicates comparisons between conditions with statistically significant differences (*p < 0.05)

Data on the looking times at the different areas (“beginning of apparatus,” “end of apparatus”) in the 30 s after the disappearance of the ball, for both experimental conditions, are shown in Fig. 5.

The independent variable condition had an effect on dogs’ looking time to the “end of the apparatus” in the 30 s after the disappearance of the ball (Wald Chi-square = 14.262, p = < 0.001, Cramer’s V = 0.983) being higher in the Depicted hole trial (estimated mean ± std. error 10.7 ± 1.1 s) than in the Real hole trial (estimated mean ± std. error 8.0 ± 1.0 s). The order of presentation, instead, did not affect the looking time at the “end of the apparatus” (Wald Chi-square = 1.604, *p* = 0.205), and the same applied to the interaction between these variables (Wald Chi-square = 1.360, *p* = 0.244). The looking time at the “beginning of apparatus” showed a non-significant effect of the condition (Wald Chi-square = 1.217, *p* = 0.270), the order of presentation (Wald Chi-square = 3.644, *p* = 0.056) and the interaction between the two variables (Wald Chi-square = 0.018, *p* = 0.892).

#### Discussion

Experiment 3 was implemented to confirm the results previously reported by Broseghini and colleagues (2024). The results showed strong consistency with the findings of the previous study: dogs looked longer at the end of the apparatus when the ball rolled over the depicted hole, compared to when it fell into it, demonstrating that the depicted hole is perceived as if it was real. These results confirm that dogs are sensitive to these pictorial cues, and that a combination of shading and linear perspective effectively elicits the perception of three-dimensionality in dogs when presented together in a two-dimensional image.

### Experiment 4 -High shading

#### Results

The “Depicted hole” trials lasted for 31.7 ± 0.1 s, and the “Real hole” trials for 31.0 ± 0.1 s. Dogs were oriented toward the ball travelling in the area “beginning of apparatus” for 89.3 ± 26.6% of the 1.0 s time. After applying a t-test, no difference in looking time towards the “beginning of apparatus” area for the two experimental conditions was found (Depicted hole condition = 0.9 ± 0.3 s; Real hole condition = 0.9 ± 0.3 s; t = − 0.294, df = 29, *p* = 0.771, Cohen’s d = −0.054).

In the Depicted hole condition, dogs were oriented towards “end of apparatus” when the ball travelled from the hole to the box for 87.9 ± 13.2% of the time.

**Fig. 6:**
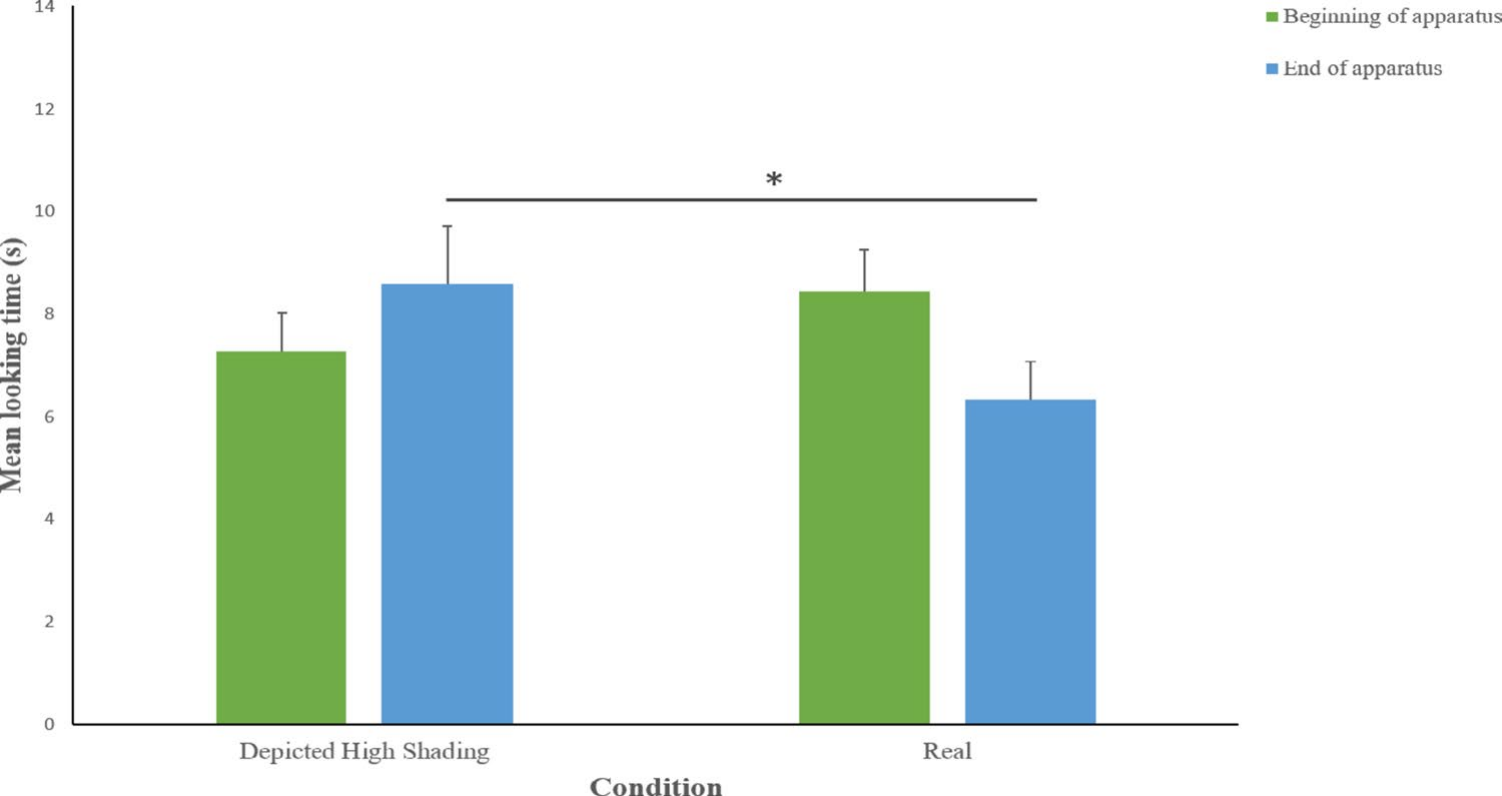
Mean looking time (± SE) paid by dogs to different parts of the experimental setting and elsewhere, in the Depicted hole with higher level of shading and Real hole conditions, during the 30 s after the ball disappeared. Line with asterisks indicates comparisons between conditions with statistically significant differences (*p < 0.05).

In Fig. 6 data on the looking times at the different areas (“beginning of apparatus,” “end of apparatus”) in the 30 s after the disappearance of the ball, for both experimental conditions, are reported.

Dogs’ looking time to the “end of the apparatus” in the 30 s after the disappearance of the ball was affected by the condition (Wald Chi-square = 7.205, *p* = 0.007, Cramer’s V = 0.983) being higher in the Depicted hole trial (estimated mean ± std. error 8.5 ± 1.1 s) than in the Real hole trial (estimated mean ± std. error 6.4 ± 0.7 s). The order of presentation did not affect the looking to the “end of the apparatus” (Wald Chi-square = 2.102, *p* = 0.147), nor the interaction between these variables (Wald Chi-square = 0.893, *p* = 0.345). The looking time at the “beginning of apparatus” for the condition (Wald Chi-square = 1.573 *p* = 0.210) was found non-significant, as well as for the order of presentation (Wald Chi-square = 0.320, *p* = 0.571), and the interaction between these variables (Wald Chi-square = 0.794, *p* = 0.373).

#### Discussion

In Experiment 4 we tested the possibility that the insufficient intensity of the shading in Experiment 2 explained the cue’s inefficacy in eliciting the perception of three-dimensionality, when presented alone. Therefore, dogs were presented with stronger shading both between the surface of the apparatus and the lateral walls of the hole and between the two lateral walls of the hole, in accordance with the direction of the light source. In this condition, dogs expressed a clear surprise reaction when seeing the ball rolling over the depicted hole, supporting that shading alone can elicit the perception of three-dimensionality in dogs, provided that it is sufficiently intense to clearly define differently oriented surfaces. Our finding is in line with previous research on non-human animals, highlighting the effectiveness of this cue across species. For instance, pigeons, chicks, chimpanzees, and even cuttlefish have demonstrated the ability to extract depth information from shading alone (Cook et al. 2012; Hershberger 1970; Imura and Tomonaga 2003; Zylinski et al. 2016). This cross-species consistency suggests that sensitivity to shading may reflect a fundamental mechanism for interpreting depth from visual input. Given that shading naturally occurs in most environments, it is not hard to think that its perception may have adaptive and evolutionary advantages.

## General discussion

In the present study we explored the relative contribution of the pictorial cues shading and linear perspective to the perception of three-dimensionality in bidimensional images in dogs. Overall, the results indicate that dogs may both need and integrate multiple pictorial cues to perceive three-dimensionality, since the cues are ineffective when presented on their own. However, three-dimensional perception can also be elicited by shading alone if presented with sufficient intensity.

Dogs’ ability to integrate multiple, individually ineffective pictorial cues to perceive three-dimensionality is intriguing. Behavioural and perceptual studies in humans have shown that the combination of multiple depth cues leads to more accurate estimations than the use of any single cue. Bülthoff and Mallot (1988) showed that shading and disparity cues lead to a stronger depth perception than shading alone and that the amount of perceived depth decreased as individual cues were removed. Not only accuracy in depth perception seems to be improved by multiple cues. Adding binocular cues to photographs of natural scenes improved the perceived depth magnitude, the reliability across repetitions, and the consistency in depth perception among individuals (Hibbard et al. 2022). The role of multiple pictorial cues seems to depend also on the specific task requirements. The weights assigned to different cues can indeed differ depending on whether the task involves perception or action (Knill 2005). Overall, the findings of this study align with Landy’s theory, stating that depth perception is often based on the interaction of multiple cues (Landy et al. 1995). However, how exactly the integration of multiple pictorial cues occurs remains an unsolved aspect in depth perception, still highly debatable, as testified by the many different proposed models of integration (Linton 2023). For instance, some Authors propose such to be explained in terms of Bayesian inference, where the reliability of each cue is taken into account to compute the most probable depth estimation (Landy et al. 1995). Other studies, however, have challenged the Bayesian model of cue combination, proposing alternative accounts based on non-probabilistic principles. The Intrinsic Constraint theory proposes that the visual system prioritizes the most stable interpretation of visual input over the most probable one, especially when faced with variations in viewing conditions (Domini 2023). The Vector Sum model does this by treating each depth cue as a part of a multi-directional signal, and the final depth perception depends on the overall strength (or length) of that combined signal (Domini 2023). Although not the aim of the present study, the integration of shading and linear perspective in our experiment could tentatively be interpreted within the framework of the Vector Sum model, where cues are combined through a vector sum rather than an average. In our case if both cues were interpretable and coherent, their vector sum would produce a perception that is deeper than what either cue alone would support.

The integration of multiple depth cues has also been investigated in the animal kingdom, showing for instance that monkeys’ perceptual accuracy increases when they can integrate stereoscopic and monocular cues (Chang et al. 2020; Schiller et al. 2011). Integration of multiple cues to three-dimensional perception extends also to other classes. For instance, pigeons are able to use both shading and linear perspective as depth cues, but their accuracy in discriminating three-dimensional objects is significantly lower when these cues are presented individually rather than combined (Reid and Spetch 1998). Moreover, a strict relationship between the number of monocular cues presented in a discrimination task and performance exists. In fact, pigeons trained to discriminate the spatial order of objects performed increasingly better as the number of available cues increased, with the best discrimination in the three-cue condition, followed by the two and one-cue conditions (Cavoto and Cook 2006). Thus, our results confirm that also a non-human animal can integrate multiple cues to achieve a more robust perception of three-dimensionality.

While the enhancement of depth and three-dimensional perception through the integration of multiple visual cues is well-documented, to the best of our knowledge only few studies proved that single depth cues no longer elicit three-dimensional perception when presented alone. Bertin and Bhatt (2006) showed that 3 months old infants did not detect any differences in the orientation of elongated cubes, promoted only by line junction. However, when shading was added to the drawings, infants successfully detected the orientation changes, suggesting that, at this age, the use of line junction information is only effective when paired with another cue (Bertin and Bhatt 2006). Other evidence shows that pictorial cues can elicit depth perception only under specific conditions: the effect of both partial occlusion and interposition was not found to be statistically significant when the figure contrast was high, but the two cues became effective when the stimuli presented low contrast, highlighting that their effectiveness is not always achieved alone (Dresp et al. 2002). In other domains of visual processing, there are examples of how the effectiveness of individual cues becomes functional when integrating multiple sources of visual information. One example is the perception of biological motion through point-light displays, where a set of dots placed on key points of a living being typically conveys no recognizable information when static, however when proper movements are added, the dots start promoting biological motion information (Johansson 1973; Brown et al. 2010; Eatherington et al. 2019). Similarly, in the Ames room illusion an observer perceives a non-distorted room when watching the scene monocularly, so basing the perception only on monocular cues. However, the illusion rapidly breaks down when binocular disparity or motion parallax cues are available, allowing the visual system to reinterpret the spatial layout more accurately (Dorward and Day 1997). So, the visual phenomena leading to a more accurate representation of the environment seem to depend not only and not always on a single cue, but rather on the integration of multiple cues. Our results align with this framework, confirming that even in dogs, the perception of three-dimensionality is favoured by the presence of multiple cues.

Although the integration of multiple cues usually promotes a more complete and accurate perceptual experience, it has been shown that, in some conditions, depth perception can also be elicited by individual cues. The study of the perception of individual depth cues by infants is broad, as the age of development of the sensitivity to pictorial cues has been an object of great interest (Kavšek et al. 2012). For instance, after 8 months of age infants perceive the relative depth ordering of surfaces thanks to interposition alone (Kavšek 2004). As for shading, four-month-old infants have been proven to process shading in the same way as adults and to be able to use the only cue of shading for detecting shapes (Imura et al. 2008). The successful role of shading in promoting three-dimensional perception has been reported also in non-human animals. Chimpanzees can perceive depth from shading at 10 months of age (Imura and Tomonaga 2003). Pigeons can discriminate the surface direction of concave and convex three-dimensional shapes by differential shading information (Cook et al. 2012). A previous study provided evidence that shading alone can trigger depth perception in chicks and that the perceptual bias toward interpreting light as coming from above is likely innate rather than learned (Hershberger 1970). Cuttlefish are sensitive to the pictorial cue of shading alone (Zylinski et al. 2016), but it has also been shown to be responsive to the individual pictorial cue of texture gradients, avoiding to swim over a surface that resembled an illusory crevasse (Josef et al. 2014). These findings collectively suggest that single pictorial cues can elicit the perception of three-dimensionality; interestingly, most studies focus specifically on shading as a primary cue. In agreement with these previous studies, our results also indicate that shading alone can successfully promote the perception of three-dimensionality in dogs. However, this seems possible only when shading reaches a sufficient intensity level. One possible explanation for the ineffectiveness of low intensity shading lies in the contrast of the presented shades. Even if clearly visible to the human eye, the contrast between the brightest and darkest areas in the hole of Experiment 2 was low and it is possible that the dog’s visual system is not sufficiently sensitive to detect it. Weber’s fraction (i.e. the minimal ratio between two intensities which grants discrimination) for dogs’ ability to discriminate brightness ranges from 0.22 to 0.27, while that of humans in the same experimental conditions ranges from 0.11 to 0.14, indicating that brightness discrimination in dogs is about twice as bad as ours (Pretterer et al. 2004). It is therefore possible that in our case the shading of Experiment 2 was presented with an intensity which was too low to promote an effective perception of three-dimensionality. However, calculated a posteriori, the Michelson contrast value between the different areas of our low intensity stimulus was found to be 3.2% (L_max_=156.8; L_min_=147), while it was previously shown that dogs’ performance significantly worsened at the 1% contrast level (De Rivera et al. 2005), making this explanation less likely. An alternative aspect that may have contributed to the different effectiveness of shading in promoting the perception of three-dimensionality is the lighting setup. When shading was presented with a low intensity, the light source was not clearly identifiable, as the room was uniformly illuminated with all ceiling lights. Conversely, in Experiment 4 the higher level of shading was achieved by illuminating the apparatus with a laterally placed spotlight, aiding in the detection of the light direction. The effectiveness of shading depends indeed on assumptions about the lighting environment. It has been shown that knowing or learning the position of the light source significantly improves the reliability of shading as a cue to infer shape, allowing the visual system to interpret shading gradients more appropriately (Harding et al. 2012). Thus, it is possible that having a clear view of the light source in Experiment 4, further increased the effectiveness of shading in dogs.

While we manipulated different characteristics of shading, such as a higher luminance contrast and a clearer light source, in the current study we did not explore the effectiveness of linear perspective under varying conditions. In fact, the role of linear perspective was only tested in Experiment 1, where it no longer elicited the perception of three-dimensionality when presented alone. Linear perspective was conveyed through high contrast alternating black and white lines converging at a vanishing point, a pattern commonly used to enhance depth perception (Gibson and Walk 1960). Dogs remained highly attentive to the apparatus also after the disappearance of the ball indicating that the ineffectiveness of the linear perspective cue was unlikely to be due to a lack of attention or low salience of the stimulus, and suggesting an ineffectiveness of perspective in eliciting three-dimensional perception by dogs. The role of linear perspective in depth perception has been studied to a limited extent in non-human animals. Gunderson and colleagues tested linear perspective on infant macaques using displays that created a depth illusion through pictorial cues. However, such displays were created using texture gradients preventing us from drawing conclusions about the individual role of the linear perspective (Gunderson et al. 1993). Horses, pigeon and baboons, were tested through a series of relative-line-length discrimination tasks and were found to be sensitive to the Ponzo or the Corridor illusion (Barbet and Fagot 2002; Fujita et al. 1991; Timney and Keil 1996). However, these illusions may not rely solely on mechanisms of depth perception and some theoretical accounts suggest that it can be explained by alternative processes such as assimilation or contrast effects (e.g., Reardon and Parks 1983; Pressey and Epp 1992). As a result, the susceptibility to these illusions alone does not conclusively demonstrate sensitivity to depth from linear perspective. Therefore, there is scarce evidence about the effectiveness of linear perspective alone in eliciting three-dimensional perception in non-human animals. Our findings suggest that dogs are not sensitive to perspective, but this aspect deserves further investigation, to disentangle whether it reflects an insensitivity to the cue itself or limitations in the way it was presented.

## Conclusion

The present study investigated the role of two monocular cues - linear perspective and shading - in promoting the perception of three-dimensionality from bidimensional images in dogs. Our results show that dogs can integrate linear perspective and shading to perceive three-dimensionality, although the same cues were ineffective when presented alone. However, high contrast shading was sufficient to elicit perception by itself. Thus, dogs’ perception of three-dimensionality entails cue integration process, and this pattern mirrors what has been observed in humans and other animals, highlighting that dogs too can combine visual cues to build spatial representations. Moreover, our finding also suggests that the perception critically depends on the intensity and clarity of the individual stimuli. Under optimal conditions, a single cue can become sufficiently salient to elicit three-dimensional perception by itself.

These findings underscore that perceptual integration strategies in dogs may have evolved to maximize efficiency in visually complex environments—such as those typical of anthropic niches. Yet, the results also suggest dogs’ ability to derive three-dimensional information from shading, which might reflect their evolutionary origin from a typically crepuscular species. Under dim light conditions, when visual spatial resolution decreases, shading patterns are more likely to remain reliable compared to other monocular cues, which require the perception of finer visual details.

These results highlight the importance of refining our theoretical understanding of dogs’ higher-level visual processes, prompting researchers to consider these aspects when designing and presenting stimuli in visual-based tasks. Future studies could apply the same methodology to investigate other pictorial cues, such as texture gradient, as the exploration of static monocular cues in dogs remains understudied. Further exploration of the role of linear perspective is also needed, in order to understand if different presentations (e.g. curved perspective or a higher saliency) could enhance the perception of three-dimensionality. Finally, future research should explore the relative contribution of evolutionary factors and environmental exposure in determining the ability of animal species to use specific depth cues. For instance, comparative studies involving crepuscular and diurnal carnivores could help determine whether sensitivity to shading is indeed an adaptive trait linked to ecological light regimes.

## Data Availability

Data are publicly available in the data repository of the University of Padua, at https://researchdata.cab.unipd.it/id/eprint/1574. Last access 29/05/25.
